# Case Report: E2 luteal phase priming in a stop GnRH agonist combined with GnRH antagonist using a delta follitropin protocol in a poor responder: clinical case

**DOI:** 10.3389/frph.2025.1661353

**Published:** 2025-10-15

**Authors:** Melissa María Morales Berrocal, Mariana Peña Miranda, Armando Miguel Roque Sánchez

**Affiliations:** ^1^ Costa Rican Department of Social Security, San José, Costa Rica; ^2^ Department of Reproductive Medicine, Latin American Network of Assisted Reproduction, CDMX, Mexico

**Keywords:** luteal estradiol supplementation, delta follitropin, poor responder, agonist stop protocol, case report

## Abstract

A 30-year-old woman with a body mass index of 20.6 kg/m² consulted due to two years of primary infertility. The patient had a history of two unsuccessful previous rounds of ovarian stimulation for IVF due to poor ovarian response. A novel ovarian stimulation approach incorporating luteal phase estradiol priming in a stop GnRH agonist plus delta follitropin-GnRH antagonist protocol was followed. *in vitro* fertilization was performed resulting in eight mature oocytes, which were fertilized and developed into two usable blasts. The patient did not achieve pregnancy from either the fresh or subsequent frozen embryo transfer. Our case demonstrates that this combined strategy (estradiol and GnRH agonist) offers dual suppression of FSH and LH, with E2 playing a critical role in preventing premature FSH surges and enhancing granulosa cell receptivity. To our knowledge, this is the first study to provide initial evidence supporting the clinical utility of combining luteal E2 priming, stop GnRH agonist and follitropin delta in this context. This case report constitutes a proof of principle that requires further studies with a large number of patients to replicate and validate the stimulation protocol.

## 1 Introduction

Controlled ovarian hyperstimulation (COH) is a critical component in the success of *in vitro* fertilization (IVF), with patient responses to exogenous gonadotropins varying widely. This variability has prompted the adoption of individualized treatment approaches. In response to ongoing challenges in managing low or poor responders, the European Society of Human Reproduction and Embryology (ESHRE) introduced standardized criteria—known as the Bologna criteria—to better define this patient population (1). According to these criteria, a poor ovarian responder is defined as a patient with two previous failed maximal stimulation attempts, in the absence of advanced maternal age or abnormal ovarian reserve tests.

Several strategies have been explored to address poor ovarian response, including the use of both gonadotropin-releasing hormone (GnRH) agonist and antagonist protocols. GnRH agonists (GnRH-ag) function by downregulating pituitary receptors and suppressing gonadotropin secretion through desensitization, whereas GnRH antagonists (GnRH-ant) act by competitively inhibiting endogenous GnRH at its receptor, leading to an immediate suppression of gonadotropin release. Orvieto et al. previously described a “stop” GnRH-ag protocol combined with GnRH-ant administration (2) as a viable option for poor responders.

In this case presentation, we introduce a novel protocol that incorporates luteal phase estradiol priming, a “stop” GnRH-ag protocol, GnRH-ant co-treatment, and a modified administration of delta follitropin. We propose that this approach may offer enhanced follicular synchronization and improved outcomes in poor responder patients.

## 2 Case report

A 30-year-old woman with a body mass index of 20.6 kg/m² consulted due to two years of primary infertility. She had been engaging in regular intercourse for two years without achieving pregnancy. She presented with regular 30-day menstrual cycles, dysmenorrhea, dyspareunia, and low back pain, with no history of previous surgeries (these symptoms suggested clinical endometriosis, but no further studies were applied). An ultrasound scan indicated a normal uterine volume and both ovaries were normal in size, with an antral follicle count of 4 and 5 (right and left, respectively). Her AMH level was 2.22 ng/mL (measured using Elecsys Cobas, Roche analyzer), while early follicular phase markers FSH 8.93 mIU/mL, LH 8.59 µIU/mL and E2 31.8 pg/mL, consistent with normal ovarian reserve markers.

The patient had a history of two unsuccessful previous rounds of ovarian stimulation for IVF. The first cycle, in August 2023 (with AMH measured at 1.83 ng/mL prior to the cycle), was initiated on day 2 of the menstrual cycle with daily subcutaneous injections of a fixed-dose combination of recombinant human FSH (r-hFSH) monotherapy at 75 IU (follitropin alfa; GONAL-f®, EMD Serono, Inc., Rockland, Massachusetts, USA), plus r-hFSH at 150 IU and recombinant human LH (r-hLH) at 75 IU (follitropin alfa and lutropin alfa in a 2:1 ratio; Pergoveris®, EMD Serono, Inc., Rockland, Massachusetts, USA) for four days. This was followed by administration of r-hFSH at 300 IU and r-hLH at 150 IU (follitropin alfa and lutropin alfa) for eight days. Due to a poor ovarian response (POR), with only two follicles >16 mm, the IVF cycle was canceled, and intrauterine insemination (IUI) was performed; however, pregnancy was not achieved.

The second IVF cycle, in October 2023, was also initiated on day 2 with a fixed-dose combination of r-hFSH monotherapy at 150 IU (follitropin alfa; GONAL-f®; EMD Serono, Inc.) and highly purified human menopausal gonadotropin (HP-hMG) at 150 IU (Menopur®, 75 IU LH:75 IU FSH; Ferring Pharmaceuticals Inc., Suffern, New York). After five days of stimulation, the cycle was canceled again due to POR, and another IUI was attempted without success.

These outcomes prompted a re-evaluation of the treatment protocol, given the hypo-response or suboptimal response to ovarian stimulation, despite ovarian reserve markers indicating normal ovarian capacity.

**Figure 1:**
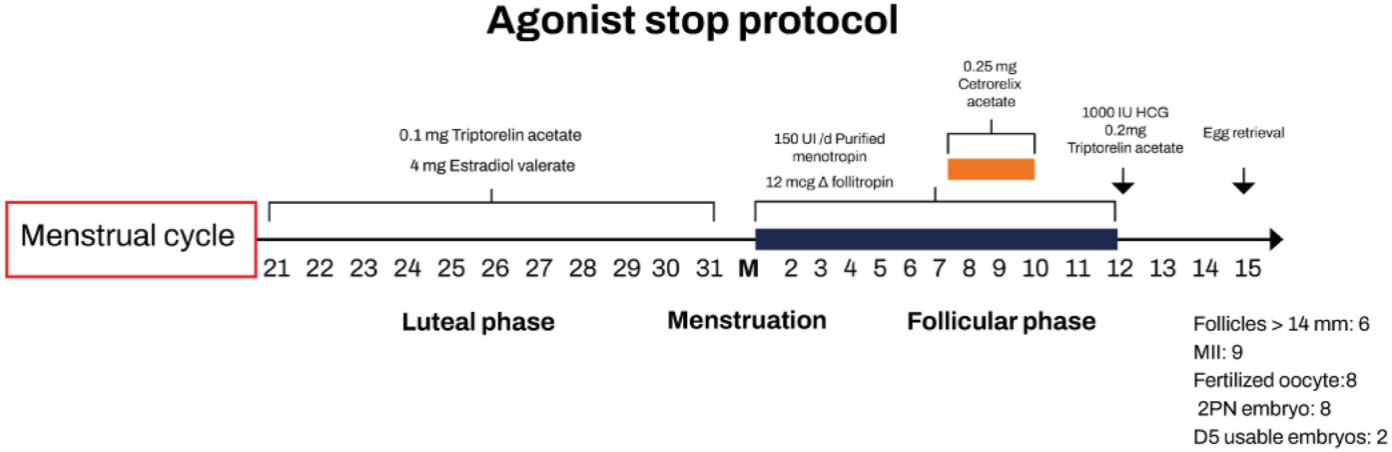
Diagram of pharmacological treatment regimen of the ovarian hyperstimulation cycle. HCG, human chorionic gonadotropin.

A third round of treatment was initiated in December 2023, using a novel strategy (Figure 1): luteal phase priming with estradiol, combined with a GnRH-ag and GnRH-ant protocol (Table 1). On day 21 of the menstrual cycle, the patient began daily administration of triptorelin acetate (Decapeptyl®; Ferring A.G., Dübendorf, Switzerland) at 0.1 mg for pituitary downregulation, along with 4 mg of oral estradiol valerate (Bayer, Germany) for luteal phase priming. Both treatments were continued until the onset of menstruation.

**Table 1:** Cycle characteristics and results in a poor responder patient.

Cycle number	Protocol	Hormon	Total gonadotropin (IU/ucg)	Days of stimulation	Serum E2 on oocyte retrieval day	Numbers of follicles (>=14 mm)	MII	Usable blastocyst	Result
1	GnRH antagonist	α follitropin + α lutropin + cetrorelix acetate	FSH (3,300 IU) + LH (1,500 IU)	13	1,050	3	ND	ND	IUI
2	GnRH antagonist	α follitropin + menotropin + cetrorelix acetate	FSH (3,000 IU) + LH (3,000 IU)	10	655	2	ND	ND	IUI
3	GnRH agonist + GnRH antagonist	Triptorelin Acetate Injection + δ follitropin + menotropin + cetrorelix acetate	FSH(1,950 IU + 156 ucg) + LH (1,950 IU)	13	1,975	6	9	2	No pregnancy

GnRH, gonadotropin releasing hormone; MII, metaphase II oocytes; D3, the third day; IU, International unit; ND, no data; IUI, intrauterine insemination; FSH, follicle stimulating hormone; LH, luteinizing hormone E2 estradiol.

Following priming, baseline laboratory results confirmed pituitary suppression:

• FSH: 3.17 mIU/mL
• LH: 5.05 µIU/mL
• Estradiol (E2): 114 pg/mL

Gonadotropin stimulation was initiated on day 2 of the cycle with fixed doses of 12 μg of follitropin delta (Rekovelle®, Ferring Inc.) plus HP-hMG 150 IU. During stimulation, the patient underwent regular transvaginal ultrasound monitoring, follicular tracking, and hormonal assessments (including estradiol, luteinizing hormone, and progesterone) to evaluate follicular development (Table 2). On day 7 of gonadotropin administration, when a follicle reached 13 mm in diameter, subcutaneous cetrorelix acetate (Cetrotide®; Asta Medica, Germany) was initiated at 0.25 mg/day as a GnRHant.

**Table 2:** Follicular dynamics (growth/endometrium) plus hormonal results on quantitative hormonal monitoring of the cycle/stimulation.

Cycle day/parameter		Day 1		Day 6		Day 10		Day 12	
Endometrial thickness (mm)		Descamative		Liquid interface		10		10	
Hormonal results	E2 (pg/ml)	114		231		1,159		1,975	
	LH (µUI/ml)	5.05		1.57		1.72		ND	
	FSH (mUI/ml)	3.12		ND		ND		ND	
	*P* (ng/ml)	0.28		0.05		0.31		1.00	
Follicle size (mm)		ND	ND	9.9 9.5 8.7 7.9 7.5 7.4	–	17.2 15.6 13 11 11 10.7 8.1 6.5	3.7	18 18 16 14 14 12 12 10 16 8	7
Right ovary	Left ovary								

ND no data; FSH, follicle stimulating hormone; LH, luteinizing hormone; E2, estradiol; P, progesterone.

Oocyte retrieval was performed 36 h after dual triggering (on cycle day 12), using 10,000 IU of hCG (Livzon Pharmaceuticals, China) and 0.2 mg of triptorelin acetate. Oocytes were retrieved via transvaginal ultrasound-guided follicular aspiration and fertilized by conventional IVF using normozoospermic sperm (seminal parameters encompasses: volume 1.7 ml, direct concentration 203 million/ml and progressive motility 48.7%). A total of nine cumulus-oocyte complexes were obtained, nine of which were mature (Metaphase II) and then got fertilized. Following embryo culture, one day-5 blastocyst grade 4Bc was transferred fresh, and one blastocyst day-5 grade 4Cc was vitrified. Three additional lower-quality blastocysts were obtained but not cryopreserved. The patient did not achieve pregnancy from either the fresh embryo transfer or the subsequent frozen embryo transfer. Vitrified blastocyst grading remained the same after thawing procedure.

## 3 Discussion

The identification of patients at risk for low oocyte yield through serum biomarkers and ultrasound imaging has become increasingly reliable (3). However, determining the most effective treatment approach for these patients remains a significant challenge in fertility clinics. An even greater challenge is posed by women who are classified as poor responders only after undergoing IVF treatment, for whom standard protocols often prove inadequate. This case represents the definition of a true deficient ovarian response (poor ovarian response POR), in which the best ovarian capacity is not reached, but may be achieved with a modification of the stimulation (3).

It is well known that estrogens work primarily in suppressing FSH secretion, so we use them as a primming since day 21 of the previous stimulation cycle. In a study (Hauzman et al.), the results showed that following the discontinuation of oral contraceptive pills (OCPs), FSH and LH levels required approximately five days to recover from significant suppression, highlighting this timeframe as an appropriate washout period before initiating stimulation in OCP-pretreated cycles (4). In contrast, estradiol (E2) pretreatment did not markedly suppress FSH levels but instead helped prevent the typical rise in FSH during the luteal-follicular transition. Furthermore, the rapid rebound of FSH observed after stopping natural estrogen suggests that only a short washout period of 1–2 days is necessary before starting stimulation (4). Chang et al. (5) theorized about a second mechanism of action of E2, in which E2 stimulates the proliferation of FSH receptors in granulosa cells.

Patients with diminished ovarian reserve or poor responders seem particularly vulnerable to the suppressive impact of pituitary desensitization protocols with OCP, which can result in a reduced number of retrieved oocytes. As a result, the use of E2 pretreatment is increasingly being explored as a strategy to improve outcomes in this group of patients (5). Chang et al. (5) showed that E2 priming provoked a more gradual and synchronized stimulation process, likely due to improved uniformity in antral follicle development.

Triptorelin acetate is a gonadotropin-releasing hormone agonist. GnRH-ag are synthetic analogs of natural GnRH. Continuous administration of GnRH-ag leads to pituitary desensitization by inducing GnRH receptor downregulation following an initial flare effect. They are widely used in controlled ovarian hyperstimulation protocols because they seem to reduce cycle cancellation rates by preventing premature LH surges and luteinization, while also enhancing follicular recruitment, leading to the retrieval of a higher number of oocytes and improved cycle management (6).

The novel combination of a GnRH-ag and estradiol (E2) priming during the luteal phase of the cycle preceding ovarian stimulation offers dual suppression of FSH and LH secretion. Estradiol exerts a negative feedback effect on the pituitary, preventing premature FSH surges while simultaneously promoting FSH receptor expression in granulosa cells. Meanwhile, triptorelin acetate, a GnRHant, induces downregulation of GnRH receptors, leading to further suppression of both FSH and LH release. This approach aims to enhance follicular synchronization, avoid the need of increasing the gonadotropin daily dose (typically associated with GnRH-ag during COH) (2) and optimize ovarian response in subsequent stimulation cycles.

Triptorelin acetate is also employed as a triggering agent. The rationale of employing triptorelin for final oocyte maturation is supported by triptorelin's half life of 4,2 h (2, 7). Since it was discontinued at the start of gonadotropin stimulation, this allows for its administration at the end of the cycle as part of a dual trigger protocol.

Follitropin delta is used also as a drug for stimulation optimization, this is a novel recombinant human FSH produced using recombinant DNA technology in a human fetal retinal cell line. Its α and β subunits have amino acid sequences identical to those of endogenous human FSH. Follintropin delta binds to specific receptors in the ovary, triggering intracellular signaling pathways that regulate the maturation of Graafian follicles and the production of estrogen by granulosa cells. Because of structural differences the novel molecule has greater systemic exposure and reduced serum clearance than follitropin alfa, leading to a stronger ovarian response than other gonadotropins (8).

The use of follitropin delta (Follitropin-Δ) has demonstrated a superior ovarian response in both normal and hyper-responder patients when compared to equivalent doses of follitropin alfa (Follitropin-α) expressed in IU (9). The recommended maximum daily dose during the first treatment cycle typically does not exceed 12 micrograms (10). Our case provides evidence supporting the use of the maximum daily dose of follitropin delta (acording the validated algorithm) in patients classified as poor responders (9). Finally, employing a mixed protocol of individualized dosing of follitropin delta and HP-hMG was previously described for optimizing the ovarian response during *in vitro* fertilization (8).

A limitation of this study is the possibility that the combined approach may have included redundant treatment. It would be interesting to analyze whether similar results could potentially have been achieved using either a GnRH agonist or estradiol priming alone with follitropin delta, without the need for combination therapy. Additionally, no genetic testing was performed on the transferred embryos.

## 4 Conclusion

To our knowledge, this is the first study to provide initial evidence supporting the clinical utility of combining luteal E2 priming, stop GnRH agonist and follitropin delta in this context. While the protocol demonstrated improved ovarian response—evidenced by increased egg retrieval—no pregnancies were achieved in either fresh or frozen transfers using embryos derived from these cycles. This suggests that while the approach may enhance follicular recruitment, its impact on embryo viability or endometrial receptivity remains unclear. Our findings highlight the need to optimize not only stimulation protocols but also subsequent transfer strategies for this patient population. Further large-scale studies are required to validate the protocol's efficacy and investigate potential confounding factors, such as embryo quality or synchronization issues, that may underlie the observed disparity between retrieval success and pregnancy outcomes.

## Data Availability

The original contributions presented in the study are included in the article/Supplementary Material, further inquiries can be directed to the corresponding author.
